# Oxidative Stress Indicators in Patients with Prostate Disorders in Enugu, South-East Nigeria

**DOI:** 10.1155/2014/313015

**Published:** 2014-04-24

**Authors:** Romanda Duru, Obioma Njoku, Ignatius Maduka

**Affiliations:** ^1^Department of Chemical Pathology, University of Nigeria Teaching Hospital, Ituku/Ozalla, Enugu state 400001, Nigeria; ^2^Department of Biochemistry, University of Nigeria, Nsukka, Enugu State 400001, Nigeria; ^3^Department of Human Biochemistry, Nnamdi Azikiwe University, Awka, Anambra State 400001, Nigeria

## Abstract

Depletion of cellular antioxidants can result from free radical formation due to normal endogenous reactions and the ingestion of exogenous substances and environmental factors. The levels of reactive oxygen species-(ROS-) scavenging enzymes such as SOD and glutathione peroxidase have been shown to be significantly altered in malignant cells and in primary cancer tissues. The aim of this study was to determine the antioxidant status of patients with prostate disorders in South-East Nigeria to ascertain the possible role of depletion of antioxidants in prostatic degeneration. 104 subjects made up of 40 PCa patients, 32 with BPH, and 32 controls participated in this study. The levels of superoxide dismutase, glutathione peroxidase, vitamin C, and vitamin E were estimated using standard procedures. The results show that both the BPH and PCa patients had a significant decrease (*P* < 0.05) in GPX, SOD, vitamin C, and vitamin E levels compared to the control subjects. However, there was also a significant decrease (*P* < 0.05) in SOD and vitamin C levels in PCa patients when compared with the BPH group. This indicates that patients with BPH and prostate cancer have decreased antioxidant status and may benefit from micronutrient supplementation.

## 1. Introduction

Benign prostatic hyperplasia (BPH) and prostate cancer (PCa) are common urologic conditions in older men which affect the quality of life. Prostate cancer is a major public health problem in developing countries where the incidence continues to increase and the mortality is still high [1]. It has been found that Africa carries an increasing cancer burden, and men of African heritage have been found to have earlier age of diagnosis of the disease and more advanced cases of the disease [2] and are almost four times more likely to die of the disease when compared to their Caucasian male counterparts [3]. Current data from most parts of the country indicate that prostate cancer is the 3rd most common cancer and the number one cause of cancer-related death [4]. Benign prostate hyperplasia (BPH) is a condition that affects as many as 62% of men aged 50 years and above [5]. Traditionally, the two conditions are considered as two distinct and unrelated diseases, although several issues suggest possible linkages. Specifically, both are hormone dependent, their incidence increases with age, and they often coexist in the same patients and are determined by a complex interaction of endogenous and exogenous factors [5]. However, there is no proven causal relationship between BPH and PCa (although both conditions may be associated with certain forms of hyperplasia), and BPH is not considered to be a premalignant lesion or a precursor of prostate carcinoma [6]. Factors such as cellular senescence, inflammation, and oxidative stress have been described as key players in the process of prostate carcinogenesis [7]. 

Oxidative stress is defined as the interruption of the balance between oxidants and reductants within the body due to excess production of peroxides and free radicals collectively called reactive oxygen species (ROS) [8]. This imbalance leads to oxidative DNA damage that constitutes an important mutagenic and carcinogenic factor in cancer pathogenesis. [9]. In living cells ROS are generated as byproducts of cellular metabolism (such as mitochondrial respiration), whereby hydrogen peroxides and superoxide anions constitute the major sources of endogenous ROS [10]. Various carcinogens may also partly exert their effect by generating ROS during their metabolism. Free radicals such as hydroxyl and alkyl radicals and other oxygen-derived species are constantly generated in vivo both by “accidents of chemistry” and for specific metabolic purposes. Also exposure to oxidant molecules from the environment (pollution, e.g., smoke, radiation, etc.) nutrition or pathologies can generate ROS [11]. Chronic increases in ROS overtime are known to induce somatic mutation and neoplastic transformation, and intracellular changes in ROS levels may lead to processes that result in cell proliferation apostasis and senescence which are associated with initiation and development of cancer including PCa [12].

To control the balance between production and removal of ROS, there are a series of protective molecules and systems globally defined as antioxidant defenses. Antioxidants which suppress such oxidative damage play important roles in aerobic organisms. They prevent free radical induced damage by preventing the formation of ROS, scavenging them or by promoting their decomposition [13]. These include enzymes such as superoxide dismutase, glutathione peroxidase, some vitamins, and metals. 

The antioxidant enzymes are said to be the body's first line of defense against ROS [14]. The enzymes work synergistically in counteracting the deleterious effect of free radicals. Superoxide dismutases (SODs) are a class of closely related enzymes present in almost all cells and in the extracellular fluids [15]. They catalyze the breakdown of the superoxide anion into oxygen and hydrogen peroxide. Superoxide dismutase enzymes contain metal ion, cofactors which can be copper, zinc, manganese, or iron depending on the isoenzyme involved. Glutathione peroxidase is an enzyme containing four selenium cofactors that catalyze the breakdown of hydrogen peroxide and organic hydroperoxides. The biochemical function of glutathione peroxidase is to reduce lipid hydroperoxides to their corresponding alcohols and to reduce free hydrogen peroxide to water [16], thus protecting the organism from oxidative damage. Glutathione peroxidase is the most abundant and is a very efficient scavenger of hydrogen peroxide.

Oxidative stress can also be assessed by measuring the plasma antioxidant vitamins. Vitamin C and vitamin E are naturally occurring free radical scavengers. Being water soluble, vitamin C is an excellent plasma antioxidant [17]. Vitamin E is classified as an antioxidant due to its ability to scavenge lipid radicals and terminate oxidative chain reactions.

Antioxidant status in Nigerian men with prostate disorders has not been fully investigated; therefore, the aim of this study is to determine the antioxidant status as indicators of oxidative stress in patients with benign prostate hyperplasia (BPH) and prostate cancer in Enugu, South-East Nigeria, and see if the value can serve as adjunct to PSA levels in diagnosis and management of patients with these prostate disorders.

## 2. Materials and Methods

### 2.1. Subjects and Study Design

One hundred and four (104) human subjects within the age bracket of 53–85 years were used in this study. They were subdivided into three main groups as follows.

*Group A*. These were apparently healthy subjects recruited from some of the employees of the Teaching Hospital who were nondiabetics, nonsmokers, and nonalcoholics. They were 32 in number and served as control subjects. They were age and sex matched with the test subjects (B and C). 

*Group B*. These were made up of 32 patients diagnosed with benign prostate hyperplasia (BPH) and were attending urology clinics at the University of Nigeria Teaching Hospital Ituku/Ozalla, Enugu. They were nondiabetics, nonsmokers, and nonalcoholics and were not taking any medication (such as lipid lowering drugs) that may interfere with the parameters.

*Group C*. These consisted of 40 prostate cancer patients who either were attending the urology clinic or were admitted at the wards in the hospital and whose clinical records were well known from their medical history. They also were nondiabetics, nonsmokers, and nonalcoholics and were not taking any medication that may interfere with the parameters.

### 2.2. Criteria for Inclusion and Exclusion

*Exclusion Criteria*. For all the groups the subjects were nondiabetics, nonsmokers, and nonalcoholics and were not taking any medication (such as lipid lowering drugs) that may interfere with the parameters.

*Inclusion Criteria*. The BPH and prostate cancer (PCa) were medically and histologically diagnosed in the Chemical Pathology and Histopathology Departments of the University of Nigeria Teaching Hospital Ituku/Ozalla, Enugu.

Data about the patients, for example, age, and so forth, was obtained from administered questionnaire and information obtained from their hospital folders. The study was conducted at the urology clinic, the wards, Histopathology and Chemical Pathology Departments of University of Nigeria Teaching Hospital (UNTH) Ituku/Ozalla, Enugu. The study was conducted on the subjects after informed consent was obtained from each subject while approval for the study was given by the ethical clearance committee of UNTH Ituku/Ozalla.

#### 2.2.1. Sample Collection and Treatment

Blood samples were aseptically drawn from an antecubital vein of subjects by trained personnel and distributed into each of evacuated tubes containing sodium citrate for plasma ascorbic acid estimation and plain centrifuge tubes for estimating total prostate specific antigen (PSA), glutathione peroxidase (GPx), superoxide dismutase (SOD), vitamin E, and ascorbic acid. All samples were protected from light while all procedures were conducted with a minimal light exposure. The samples in the plain tubes were allowed to clot. All samples were spun at 4,000 rpm, for 10 minutes in a Jenlab bench centrifuge, model 80-2, and the sera pipetted into serum bottles and analyzed. The plasma for the ascorbic acid was separated from packed cells after the samples were spun.

### 2.3. Laboratory Methods

Total PSA concentration was determined using solid phase two-site immunoassay (ELISA) method of Stowell et al. [18] using AccuDiag—PSA ELISA kits from Diagnostic Automation/Cortez Diagnostics, Inc., USA. Glutathione peroxidase activity was determined by the method of Paglia and Valentine, [19] using EnzyChrom Glutathione Peroxidase (GPx) assay kits (EGPX-100) from BioAssay Systems, USA. Superoxide dismutase activity was determined by the method of Ukeda et al. [20] using EnzyChrom Superoxide Dismutase assay kit (ESOD-100) from BioAssay Systems, USA. Vitamin C was determined by the method of Nino and Shah [21] while vitamin E estimation was according to the method described by Fabianek et al. [22]. All chemicals used in this study were of the analytical grade and products of May and Baker, England, and Sigma-Aldrich Corporation, USA. To monitor and ensure the reproducibility and accuracy of the analytical techniques, control samples, reagent blanks, and known samples were interspersed with the test samples.

## 3. Data Analysis

Statistical Package for Social Sciences (SPSS), version 17, was used for data analysis. Results were expressed as mean ± standard deviation and tests of statistical significance were carried out using one-way analysis of variance (ANOVA). Statistical significance was defined as *P* < 0.05. Correlation coefficient between analytes was calculated using Pearson correlation coefficient at 95% and 99% confidence interval.

## 4. Results

Table 1 shows the mean levels of PSA in the BPH, PCa, and control groups (A, B, and C). The PSA levels in PCa group (54.9 ± 36.8) were significantly increased (*P* < 0.05) compared to both the BPH (8.1 ± 9.0) and control (2.8 ± 2.8) groups. The BPH also showed a significant increase (*P* < 0.05) in PSA levels when compared with the control group but the levels were not as high as in PCa.

Table 2 shows the mean levels of glutathione peroxidase (GPx), superoxide dismutase (SOD), vitamin E, and vitamin C in the different groups (A, B, and C). The PCa group showed a significant decrease (*P* < 0.05) in SOD (48.9 ± 32.7) and GPx (1166.9 ± 998.6 U/L) concentrations compared to the control group (GPx: 2984 ± 1668 U/L; SOD: 167.4 ± 71.3). The decrease in BPH group (GPx: 1385 ± 1133; SOD: 75.2 ± 51.4) was also significant (*P* < 0.05). Also the SOD levels in PCa group were significantly decreased (*P* < 0.05) compared to the BPH group. The levels of antioxidant vitamins (vitamins C and E) were significantly decreased (*P* < 0.05) in PCa (0.4 ± 0.2 mg/dL and 5.2 ± 1.9 *μ*g/mL, resp.) and BPH (0.6 ± 0.4 mg/dL and 7.0 ± 2.4 *μ*g/mL, resp.) when compared with the control group (1.3 ± 0.8 and 14.2 ± 9.1 *μ*g/mL, resp.). Also the PCa group showed a significant decrease (*P* < 0.05) in the vitamin C concentration compared to the BPH group. However, compared to the BPH group, the decrease in vitamin E was not significant.

**Figure 1:**
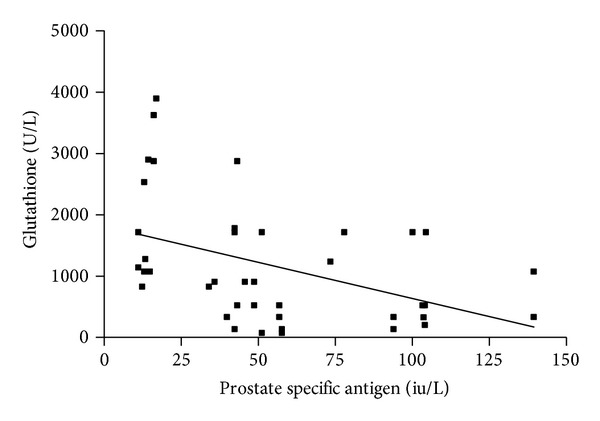
Correlation between glutathione peroxidase and PSA in PCa. The figure shows that there is a significant negative correlation between prostate specific antigen and glutathione peroxidase (*r* = −0.4346, *P* = 0.0051) in the prostate cancer subjects.

**Figure 2:**
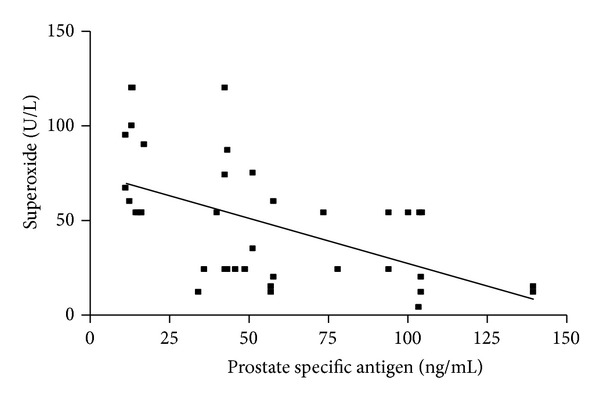
Correlation between serum superoxide and PSA in PCa subjects. The figure shows that there is a highly significant negative correlation between prostate specific antigen and superoxide dismutase (*r* = −0.5367, *P* = 0.0004) in the prostate cancer subjects.

**Figure 3:**
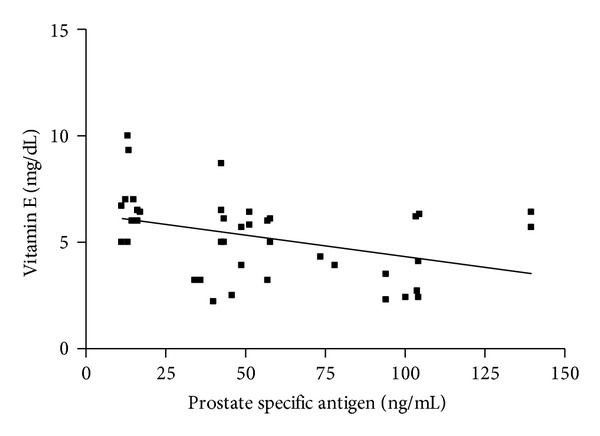
Correlation between vitamin E levels and PSA in PCa subjects. The figure shows that there is a significant negative correlation between prostate specific antigen and vitamin E levels (*r* = −0.3890, *P* = 0.0131) in the prostate cancer subjects.

Table 3 and Figures 1, 2, and 3 depict the relationship between serum glutathione peroxidase, superoxide dismutase, and vitamins C and E and PSA in prostate cancer group. There is significant negative correlation between PSA and vitamin E (*r* = −0.3890,  *P* = 0.0131) (Figure 3), but there is highly significant negative correlation between PSA and glutathione peroxidase and superoxide dismutase (*r* = −0.4346,  *P* = 0.0051; and *r* = −0.5367,  *P* = 0.0004, resp.) (Figures 1 and 2). With regard to BPH patients, no correlation was found between glutathione peroxidase, superoxide dismutase, and vitamin E and PSA. There was no significant relationship (*P* > 0.05) between PSA and vitamin C in all the subjects. 

Table 4 shows multiple linear regression between PSA and antioxidants studied in the different subjects. The result shows similar relationship between PSA and the antioxidants studied as shown in Table 3.

## 5. Discussion

**Figure 4:**
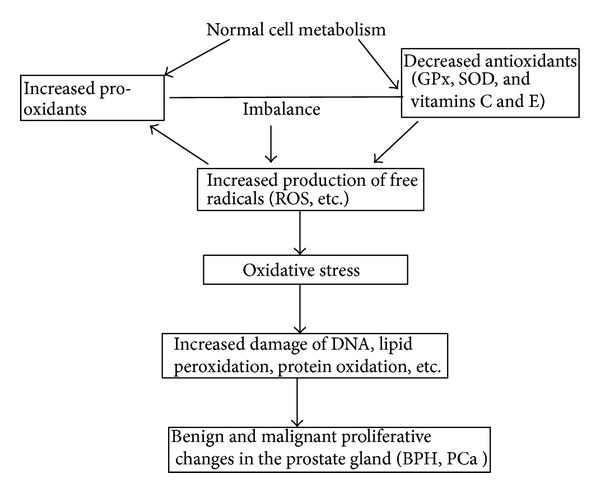
Schematic representation of the relationship between prooxidants and antioxidants in prostate disorders. An imbalance between the formation of reactive oxygen species (ROS) and the antioxidant defence capacity due to the depletion of the antioxidant system results in oxidative stress and causes increased damage of DNA, lipid peroxidation, and protein oxidation.

We undertook this study to determine some oxidative stress indicators, namely, antioxidant enzymes (glutathione peroxidase and superoxide dismutase) and vitamins (vitamin C and vitamin E) in the patients with benign prostate hyperplasia and prostate cancer. Our results show that the antioxidant levels of both enzymatic (SOD and GPX) and nonenzymatic (vitamins C and E) parameters were significantly decreased (*P* < 0.05) in the prostate cancer subjects when compared with the controls and this correlated inversely with high PSA values. This inverse relationship indicates that oxidative stress is positively associated with high PSA levels. This agrees with Akinloye et al. [23] who reported that antioxidant levels, both enzymatic and nonenzymatic, were significantly reduced (*P* < 0.05) in subjects with high PSA values. Many biochemical studies have reported that SOD is lowered in most types of primary cancers and cancer cell lines. Studies on antioxidant enzymes in human lung, renal, and prostate cancers confirmed the reduced levels of this enzyme in various cancers [23, 24]. In a recent study, Sandhya et al. [25], working in India, found that SOD levels were lower in prostate cancer patients than in those without the disease. According to Barrera et al. [26] the levels of reactive oxygen species- (ROS-) scavenging enzymes, SOD and glutathione peroxidase, have been shown to be significantly altered in malignant cells and in primary cancer tissues. The current finding that the activities of the antioxidant enzymes (SOD and GPx) decreased significantly (*P* < 0.05) in PCa patients when compared with control subjects is also consistent with earlier studies by Aydin et al. [27] who reported that the levels of glutathione peroxidase and superoxide dismutase were decreased in prostate cancer. Similarly, Kotrikadze et al. [28] reported decreased levels of SOD in patients with PCa while Woźniak et al. [11] reported decreased GPx activity in the erythrocytes of PCa patients. Furthermore, decreased level of GPX in PCa has been observed to be correlated with elevated levels of thiobarbituric acid reacting substances [24], thus indicating its involvement in lipid peroxidation. Depletion of cellular antioxidants can result from free radical formation due to normal endogenous reactions and the ingestion of exogenous substances and environmental factors. However, our findings differ from Yeh et al. [29], Surapaneni and Venkata [30], and Battisti et al. [31] who observed significantly higher superoxide dismutase (SOD) levels in patients with carcinoma of prostate. These researchers attributed the rise in the levels of SOD in their study subjects to its induction to counter the effect of increased oxidative stress. We also observed significantly lower levels of GPx and SOD in BPH patients compared to the control subjects. This is consistent with the findings of Srivastava and Mittal [32] who reported significantly decreased GPx activity in BPH patients. The decreased levels of these antioxidant enzymes indicate that there is significant alteration of prooxidant and antioxidant status in BPH and prostate cancer patients showing that oxidative stress is implicated in etiology of these disorders (Figure 4). The negative correlation between serum PSA and GPx observed in prostate cancer patients indicates the generation of more free radicals and hence oxidative stress which leads to the destruction of protein structure or formation of DNA adducts. These cascades of events may lead to reduced expression of the detoxifying enzymes or protein, which can promote development of prostate cancer (Figure 4).

It was also observed in this study that the antioxidant vitamins, namely, vitamins C and E, were significantly lower in both BPH and PCa subjects compared to the control subjects. This is similar to the results of Sandhya et al. [25] who reported decreased levels of nonenzymatic antioxidants, namely, vitamins C and E, in the plasma and erythrocytes of prostate cancer patients compared to normal subjects. Vitamin E functions as an antioxidant that scavenges lipid radicals and terminates oxidative chain reactions by interacting with the lipid peroxyl radical, preventing it from generating a new radical and perpetuating the chain reaction by oxidizing other lipids [30]. Vitamins C and E act synergistically to protect lipids and lipid structures against peroxidation. In addition, vitamin C regenerates vitamin E thereby permitting vitamin E to function again as a free radical chain breaking antioxidant. Therefore depletion of these antioxidant vitamins can lead to generation of lipid peroxides in erythrocyte membranes resulting in subsequent neoplastic transformation (Figure 4).

## 6. Conclusion

This study shows that antioxidant levels are decreased in benign prostate hyperplasia and prostate cancer patients. The resulting oxidant-antioxidant imbalance suggests that patients with prostate disorders are exposed to a lot of oxidative stress which may be one of the factors responsible for the development of BPH and prostate cancer. Assessing the antioxidant levels in the patients with these prostatic disorders may assist in their proper management and reducing disease morbidity. Thus, there may be need for antioxidants supplementation in the management of these patients since antioxidant deficiency may be associated with more cellular degeneration, cancer progression, and poor prognosis.

## Figures and Tables

**Table 1:** Mean levels of PSA in the different groups (A, B, and C).

Groups	PSA ng/mL
A *n* = 32	2.8 ± 2.8
B *n* = 32	8.1 ± 9.0^a^
C *n* = 40	54.9 ± 36.8^ab^

A: normal control group; B: BPH group; C: PCa group; *n*: number of subjects.

^a^ *P* < 0.05 when compared with group A. ^b^ *P* < 0.05 when compared with group B.

**Table 2:** Mean levels of glutathione peroxidase (GPX), superoxide dismutase (SOD), vitamin E, and vitamin C in the different groups (A, B, and C).

Groups	GPX (U/L)	SOD (U/L)	Vitamin C (mg/100 mL)	Vitamin E (µg/mL)
A *n* = 32	2984 ± 1668	167.4 ± 71.3	1.3 ± 0.8	14.2 ± 9.2
B *n* = 32	1385 ± 1133^a^	75.2 ± 51.4^a^	0.6 ± 0.4^a^	7.0 ± 2.5^a^
C *n* = 40	1166.9 ± 998.6^a^	48.9 ± 32.7^ab^	0.4 ± 0.3^ab^	5.2 ± 1.9^a^

A: normal control group; B: BPH group; C: PCa group; *n*: number of subjects.

^a^ *P* < 0.05 when compared with group A. ^b^ *P* < 0.05 when compared with group B.

**Table 3:** Correlation between PSA and SOD, GPx, and vitamin E in BPH and PCa.

Parameter	Control		BPH		PCa	
	*r*	*P* value	r	*P* value	r	*P* value
GPx	−0.1887	0.3007	0.0442	0.8100	−0.4346	0.0051**
Superoxide	0.1215	0.5076	0.2323	0.2007	−0.5367	0.0004**
Vitamin C	0.0848	0.6444	−0.1471	0.4216	0.2498	0.1200
Vitamin E	0.1966	0.2807	−0.1318	0.4720	−0.3890	0.0131*

*There is a significant negative correlation.

**There is highly significant negative correlation.

**Table 4:** Multivariate analysis of oxidative stress indicators in the control, BPH, and prostate cancer subjects.

Variables	Control (*n* = 32)		BPH (*n* = 32)		PCa (*n* = 40)	
	*R* ^2 ^ (95% CI)	*P* value	*R* ^2^ (95% CI)	*P* value	*R* ^2^ (95% CI)	*P* value
GPx	0.03562	0.3009	0.001957	0.8100	0.1889	0.0051**
SOD	0.01477	0.5076	0.05398	0.2007	0.2881	0.0004**
Vitamin C	0.007193	0.6444	0.02165	0.4216	0.06241	0.1200
Vitamin E	0.03867	0.2807	0.01738	0.4720	0.1513	0.0131*

*P < 0.05; **P < 0.01 at 95% confidence interval and 30 and 38 degrees of freedom (for *n* = 32 and *n* = 40, resp.).
